# Recognition and Management of Cutaneous Polyarteritis Nodosum Versus Livedoid Vasculitis: A Case Report

**DOI:** 10.7759/cureus.6788

**Published:** 2020-01-27

**Authors:** Jessica Forbes, Milad Heydari, Marc M Kesselman, Miguel Villacorta

**Affiliations:** 1 Dermatology, Dr. Kiran C. Patel College of Osteopathic Medicine, Nova Southeastern University, Davie, USA; 2 Rheumatology, Dr. Kiran C. Patel College of Osteopathic Medicine, Nova Southeastern University, Davie, USA; 3 Dermatology, Broward Health Medical Center, Delray Beach, USA

**Keywords:** polyarteritis nodosom, livedoid vasculitis, stellate scarring, vasculitis, cpan, cutaneous polyarteritis nodosum, atrophie blanche

## Abstract

Cutaneous polyarteritis nodosum (CPAN) is a vasculitis of small and medium-sized muscular arteries of the dermis and subcutaneous tissue with no associated systemic involvement. A common presentation of CPAN can be misinterpreted as a non-invasive form of livedoid vasculitis, synonymous with the “atrophie blanche” which similarly presents as ivory-white stellate-shaped scars. Although hyperpigmentation can also be present, as seen in our 47-year-old female patient, cutaneous polyarteritis nodosum is unique due to the etiology of the inflammatory illness which requires a deep, segmented skin biopsy for diagnosis in order to identify the vessel inflammation. In this case report, we discuss a patient with a 20-year history of painful, recurrent ulcerations and polyneuritis with previous ulcer eruptions that healed as ivory-white stellate scarring. AB cutaneous forms of polyarteritis nodosum (PAN) may be only one manifestation of the disease, with other presentations in association with multi-organ system disease. This report will discuss the necessity of a high index of clinical suspicion with a clinical presentation similar to that of our patient. We will discuss the importance of early recognition and diagnosis of cutaneous vasculitis, such as CPAN, based on clinical presentation and history in hopes of limiting morbidity and the risk of progression to systemic forms of the disease.

## Introduction

Cutaneous polyarteritis nodosum (CPAN) is a vasculitis affecting the small to medium-sized muscular arteries of the dermis and subcutaneous tissue without visceral involvement [1]. It was first described by Lindberg in 1931 as a variant of systemic polyarteritis nodosum [2]. CPAN typically presents as painful, subcutaneous nodules or ulcerations of the lower extremities. Diagnosis of this entity can be challenging as healed lesions present as ivory-white, stellate-shaped scars, also known as “atrophie blanche,” which are often misdiagnosed as a non-invasive form of livedoid vasculitis [1]. The diagnostic technique to confirm livedoid vasculitis includes a superficial punch biopsy of a new ulceration identifying hyalinosis, intraluminal thrombosis, and endothelial proliferation [3]. With histopathologic evidence of livedoid vasculitis, recommended therapies focus on anticoagulation as monotherapy [3]. However, identification of fibrinoid necrosis in combination with a neutrophilic infiltrate and extravasation of red blood cells by a deep, segmental biopsy necessitates a much different approach to the management of CPAN which conservatively begins with nonsteroidal anti-inflammatory drugs (NSAIDs) and then immunosuppressive therapy [4].

## Case presentation

**Figure 1:**
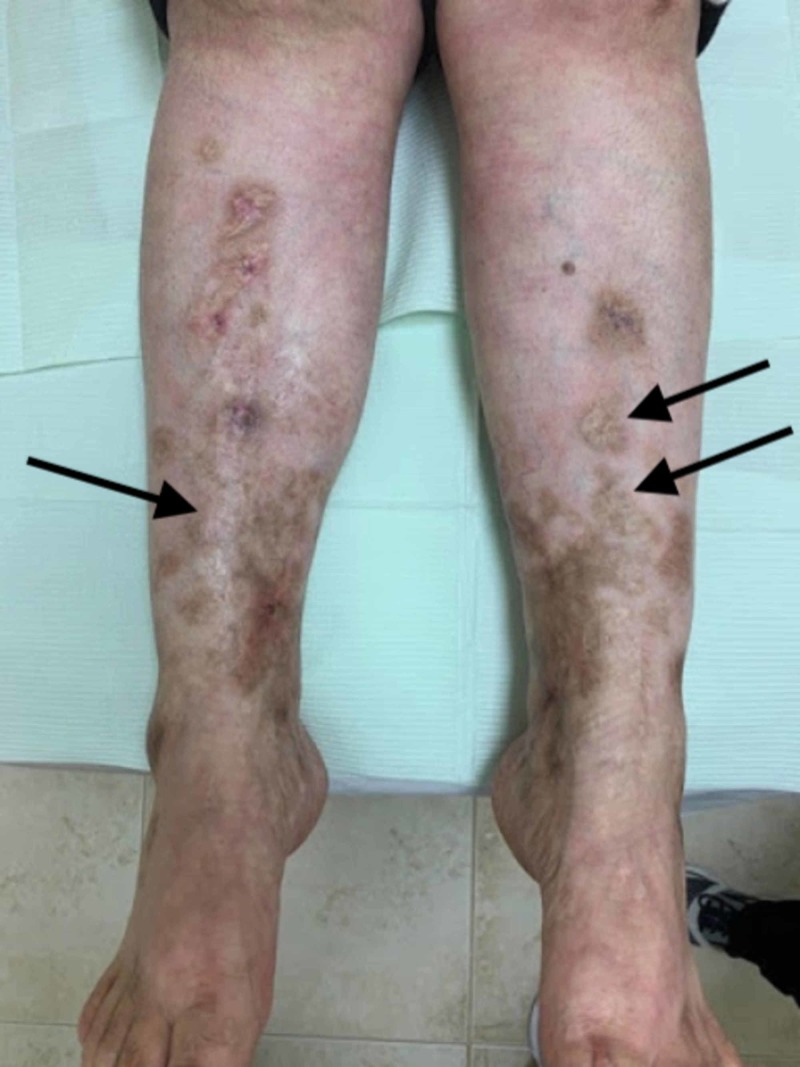
Bilateral lower extremities showing stellate scarring with atrophie blanche

**Figure 2:**
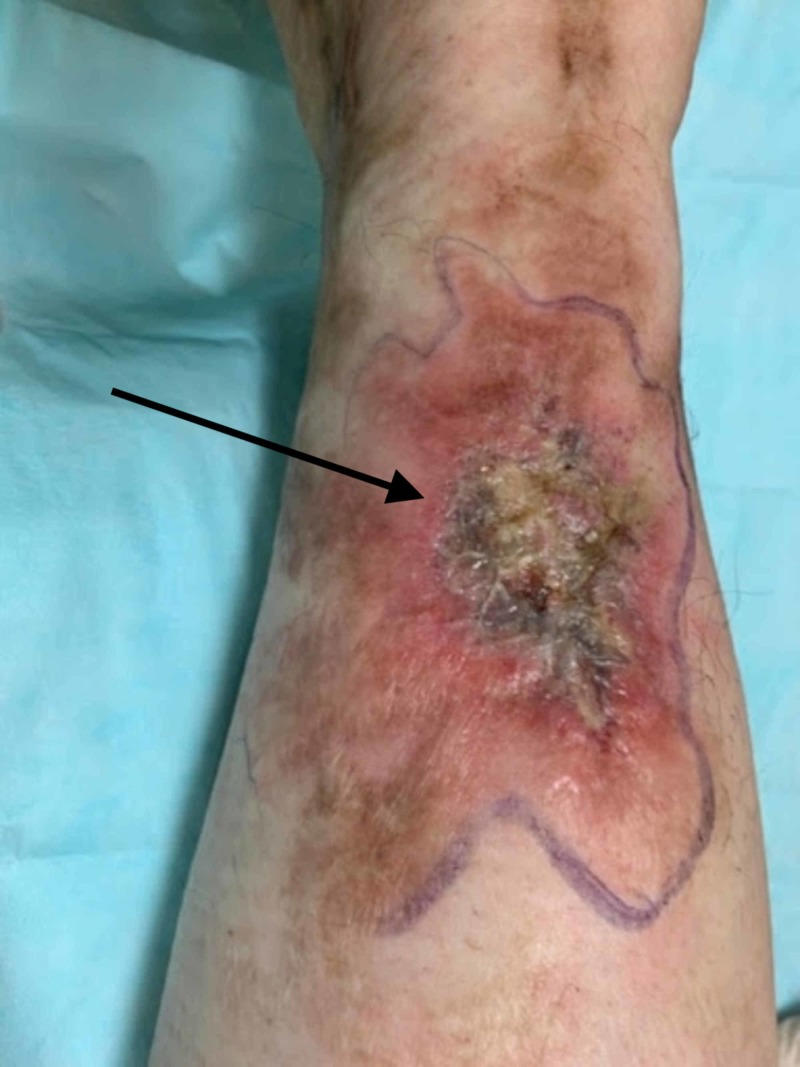
A new 3 cm ulceration on the posterior lower leg with surrounding erythema

A 47-year-old Hispanic female presented with a flare of painful ulcers on the left ankle and shin with hyperpigmentation. She complained of new-onset of distal, radiating pain of the left thigh and distal paresthesia surrounding the cutaneous, painful ulcers which she had been managing with bleach/vinegar soaks and Vaseline. The patient reported a 20-year history of similar flares and livedoid vasculitis, atrophie blanche type. On physical examination, the patient had reticulated patches on the bilateral upper and lower extremities with stellate scarring in an ivory-white pattern (Figure 1) and a new 3 cm ulcer with superficial crusting and surrounding erythema (Figure 2). The patient had been treated with trials of various medications, including prednisone, Imuran, Enbrel, Cytoxan, Plaquenil, Lovenox, and dapsone, and endured continuous relapses of ulcerations. Testing for C3/C4/ANCA, anti-centromere antibody were all negative. Histopathologic examination of the patient’s medial leg biopsy demonstrated a superficial and deep perivascular infiltrate with eosinophils, neutrophils, and focal disruption of the vascular architecture. The presence of the mixed infiltrate composed predominantly of neutrophils and few eosinophils in combination with vessel inflammation yielded a highly suspicious diagnosis of cutaneous polyarteritis nodosa.

## Discussion

Since previous ulcerations can often heal as ivory white stellate shaped scars, accurate diagnoses of CPAN may be challenging and often misdiagnosed as livedoid vasculitis [5]. However, proper disease recognition and diagnosis may foster more efficient pharmacologic management, including the prompt use of corticosteroids and other adjuvant immunosuppressant therapies such as cyclophosphamide, rather than initiating anti-platelet and or fibrinolytic therapy, perhaps decreasing the severity of exacerbations and risk of progression to systemic PAN [1, 3]. Another therapeutic goal for CPAN patients is pain management and prevention of further ulceration, in which direct oral anticoagulants (DOACs), including rivaroxaban monotherapy, effectively reduced both without relevant side effects in some patients [6]. Patients presenting with refractory disease and atrophie blanche lesions are often placed on a myriad of pharmacologic trials and advised smoking cessation, avoid irritants, begin compression therapy, elevate distal extremities, and have basic wound care readily available [7]. Our patient's extensive medical management included previous trials of multiple high-dose steroid regimens, even cyclophosphamide, over a 20-year period. Due to the severe and painful recurrence of symptoms, our patient was previously treated at a wound care center and responded well with levofloxacin. Her 20-year history of relapsing and recurring vascular complications and ulcerations provides a unique case that is relevant to the management of vascular complications of many other chronic, refractory disease states. To approach her refractory symptoms, we discussed trials of multiple off-label drugs, including rituximab, Cytoxan, and mycophenolate mofetil. The challenge with managing her case was confounded by patient compliance issues and the severity of her refractory disease. However, the patient was placed on a trial of mycophenolate mofetil, 1.5 g PO BID, prednisone 30 mg PO QD, levofloxacin, 750 mg QDx one week, vitamin D, calcium, Dakin's solution soaks for acute ulcers, and recommended follow-up in one month. Due to her prolonged unresolved symptoms, an additional trial of cyclophosphamide infusion and the use of rituximab were also discussed. The patient treatment plan was reevaluated at her next follow-up appointment, where patient compliance with the previously prescribed regimen was also in question. This case exemplifies the importance of early diagnosis, medication review, close follow-up, and patient compliance; individually, each may greatly contribute to the development of severe vascular complications and quality of life.

Prolonged severe ischemia in the skin and hypoxia are thought to stimulate angiogenesis in the surrounding tissues and recruitment of new dermal blood vessels, promoting ulcer formation [8]. Other chronic diseases, such as peripheral vascular disease, may resemble CPAN and other vasculitic conditions due to suspected similar mechanisms and phenotypic mimicry [9]. Since many diseases may end-stage to atrophie blanche, such as hydroxyurea-related ulcers, sickle cell disease, and anti-phospholipid antibody syndrome, the differential diagnoses may be distinguished with the fact that these other disorders lack a history of previous punched-out ulceration [10]. Potential complications significantly differ between livedoid vasculitis and CPAN; accurate differentiation of the etiology would perhaps promote more effective management of care [5]. Early recognition of this disease process could also lessen the associated morbidity, allowing efficient monitoring and limitations of the disease progression [5]. Cutaneous polyarteritis nodosum has also been linked to the history of Group A b-hemolytic streptococcus infection, hepatitis B infection, and in association with sequelae of adverse effects from medications [4]. A high index of suspicion and evidence-based diagnosis from clinical presentation of chronic, relapsing ulceration with a background of hyperpigmentation and atrophie blanche is essential due to necessary routes of management to control CPAN and aid in preventing unnecessary severe outcomes. 

## Conclusions

With a chronic clinical course involving several relapses, this disease is likely immune complex-mediated and does accompany a more favorable prognosis than the systemic form. Mild cases may be managed with nonsteroidal anti-inflammatory drugs, but as disease severity progresses, corticosteroids and adjuvant therapy are generally used to achieve a response. Cutaneous polyarteritis nodosum has been linked to other diseases and infectious processes, such as hepatitis B, Group A b-hemolytic streptococcus, and inflammatory bowel disease. With a wide range of etiologies, and often phenotypically similar in presentation, CPAN is often challenging to manage. With a high index of suspicion, early recognition of the disease process by dermatologists and rheumatologists can result in satisfactory management of patients with cutaneous polyarteritis nodosum. Routine follow-up for patients is recommended twice yearly with asymptomatic periods and more often during exacerbations to rule out progression to systemic PAN, although this is rare.
